# Effects of Dietary Gallic Acid on Growth Performance, Meat Quality, Antioxidant Capacity, and Muscle Fiber Type-Related Gene Expression in Broiler Chickens Challenged with Lipopolysaccharide

**DOI:** 10.3390/ani14243670

**Published:** 2024-12-19

**Authors:** Taidi Xiong, Zhilong Chen, Mubashar Hassan, Cui Zhu, Junyan Wang, Shujun Tan, Fayuan Ding, Zhonggang Cheng, Jinling Ye, Qiuli Fan, Danlei Xu, Shouqun Jiang, Dong Ruan

**Affiliations:** 1 Institute of Animal Science, Guangdong Academy of Agricultural Sciences, State Key Laboratory of Swine and Poultry Breeding, Key Laboratory of Animal Nutrition and Feed Science in South China, Ministry of Agriculture and Rural Affairs, Guangdong Provincial Key Laboratory of Animal Breeding and Nutrition, Guangzhou 510640, China; abxtd1997@163.com (T.X.); folder737497@163.com (Z.C.); mubashar.hassan@webmail.hzau.edu.cn (M.H.); 18799050298@163.com (J.W.); tanshujun_jun@163.com (S.T.); dingfayuan@163.com (F.D.); chengzhonggang@yeah.net (Z.C.); yejl2014@163.com (J.Y.); fanqiuli_829@163.com (Q.F.); 2 School of Animal Science and Technology, Foshan University, Foshan 528225, China; juncy2010@gmail.com; 3 Binhai Agricultural College, Guangdong Ocean University, Zhanjiang 524000, China; 4 Rensselaer Polytechnic Institute, Troy, NY 12180, USA; xud6@rpi.edu

**Keywords:** gallic acid, broiler, lipopolysaccharide, growth performance, meat quality, antioxidant capacity

## Abstract

In this study, broilers were selected as the research object to investigate the effects and mechanisms of dietary gallic acid (GA) supplementation on growth performance, meat quality, antioxidant capacity, and muscle fiber-related gene expression. A total of 750 one-day-old healthy 817 male crossbred broiler chickens were divided into five treatment groups, with six replicates per group. Birds in the control (CON) group and LPS-challenged treatment (LPS) group were fed a basal diet, and birds in the other three treatment groups received the basal diet with 150, 300, or 450 mg/kg added GA (GA150, GA300, GA450). On days 14, 17, and 20, chickens in the LPS, GA150, GA300, and GA450 groups received intramuscular injections of LPS, while chickens in the CON group received saline. The results showed that the addition of GA to the diet could effectively increase the average daily gain (ADG) of broilers from 1 to 50 days of age, and had a trend (*p* = 0.078) of increasing the average daily feed intake (ADFI). Adding 450 mg/kg GA to the diet significantly reduced (*p* < 0.05) the drip loss and pH value of pectoral muscles 45 min after slaughter, and significantly increased (*p* < 0.05) the lightness value of pectoral muscles 45 min post-slaughter. With an increase in GA level, the content of total volatile basic nitrogen (TVB-N) in pectoral muscles decreased linearly (*p* < 0.05), and the concentration of C22:6n-3 increased linearly (*p* < 0.05). GA effectively improved (*p* < 0.05) the antioxidant capacity of muscles and significantly increased (*p* < 0.05) the activity of total superoxide dismutase (T-SOD) in pectoral muscles after LPS stimulation, exhibiting linear and quadratic changes (*p* < 0.05). It also significantly increased (*p* < 0.05) the activity of hydrogen peroxide and decreased the activity of glutathione peroxidase (GSH-Px), while it linearly decreased (*p* < 0.05) the content of malondialdehyde (MDA). In addition, the dietary supplementation of GA significantly increased (*p* < 0.05) the expression levels of myosin heavy chain (*MyHC*) *I* and *MyHC IIa* in pectoral muscles and significantly decreased (*p* < 0.05) the expression level of *MyHC IIx*. In summary, the dietary addition of GA can alleviate the effect of the stress response on the growth performance of broiler chickens and improve antioxidant capacity and meat quality. The appropriate amount of dietary GA at each stage was 300 mg/kg.

## Simple Summary

Gallic acid (GA) is a type of phenolic acid which is widely found in Chinese herbal medicines and fruits. It has strong antioxidant and antibacterial properties. However, there are few reports on the research on and application of GA’s antibacterial, anti-inflammatory, and antioxidant functions in animal production. This experiment aims to explore the effects of dietary GA on growth performance, antioxidant capacity, and meat quality in 817 crossbred broilers after lipopolysaccharide (LPS) stimulation. The study found that the dietary addition of GA improved growth performance, increased the proportion of polyunsaturated fatty acids and the antioxidant capacity of pectoral muscles, and promoted the differentiation of muscle fibers from heavy-chain types to oxidative muscle fibers. Considering the growth performance and meat quality indicators, an appropriate level of dietary GA supplementation at each stage is 300 mg/kg.

## 1. Introduction

In the poultry industry, antibiotics have been widely used as growth promoters with a sub-therapeutic dosage due to their outstanding efficacy in feed conversion and animal growth [1]. However, as people’s requirements for animal food safety and health are rapidly increasing, the use of antibiotics as preventive and therapeutic drugs in poultry production has been increasingly questioned. The blind and irregular use of antibiotics will not only lead to the emergence of bacterial resistance and resistant strains, but also cause antibiotic residues in chicken meat and eggs, directly endangering the health of consumers [2]. Additionally, stressors such as pathogens and toxins pose a significant threat to the poultry industry. Under immune stress, the overproduction of inflammatory cytokines can lead to damage in intestinal structure and barrier function, thereby affecting the digestion and absorption of nutrients and increasing the risk of disease [3]. Lipopolysaccharide (LPS), a component of the cell wall of Gram-negative bacteria, can cause severe oxidative stress, induce inflammation, change the distribution of nutrients, reduce growth potential, and induce various diseases [4,5]. It is widely used to build stress models [6,7]. Therefore, the search for feed additives with antimicrobial activity and other effects that improve the general health of animals and reduce the need for antibiotics contributes to the safeguarding of animal welfare and the improvement of livestock productivity, as well as to the improvement of consumer health [8]. 

Gallic acid (GA), also known as 3,4,5-trihydroxybenzoic acid, can be obtained through the acidic hydrolysis of tannins and possesses strong antioxidant and anti-inflammatory properties, showing great potential in animal production [9]. A previous study showed that GA effectively inhibited inflammation and oxidation in vitro and in vivo and altered metabolic and bacterial profiles in a colitis model [10]. Starčević et al. found that adding 5 g/kg GA to the diet of Ross chickens increased the plasma n-3 polyunsaturated fatty acid content and pectoral muscle fat deposition of 35-day-old Ross chickens, and also reduced liver cholesterol levels [11]. Another study found that the total phenolic content in the thighs of 35-day-old Ross chickens whose diets were supplemented with a mixture of 1% GA and linseed oil (1:1) significantly increased and reduced the volatile basic nitrogen content of pectoral muscles on the seventh day of storage. At the same time, the bacterial abundance in thigh muscles increased slower than that in the control group during storage, indicating that chickens fed with a GA-supplemented diet were less susceptible to spoilage and were more durable in storage [12]. However, there is currently a lack of research on the effects and mechanisms of GA on improving broiler chicken quality. 

Consequently, the aim of this study was to elucidate the effects of dietary GA on the growth performance, muscle quality, and antioxidant capacity of broiler chickens after LPS stimulation, and to provide a basis for the application of its products in broiler production.

## 2. Materials and Methods

### 2.1. Animals and Experimental Design

The experimental protocol was approved by the Animal Care Committee of the Institute of Animal Science, Guangdong Academy of Agriculture Science, Guangzhou, P. R. China, with the approval number GAASISA-2022-003.

A total of 750 1-day-old male 817 crossbred broilers (39.50 g ± 0.20 g) were randomly assigned to 5 treatment groups (Table 1), each with 6 replicates of 25 broilers. Birds in the control (CON) group and LPS-challenged treatment (LPS) group were fed a basal diet, and birds in the other 3 treatment groups received the basal diet with 150, 300, or 450 mg/kg added GA (GA150, GA300, GA450). On days 14, 17, and 20 of the trial, broilers in the LPS, GA150, GA300, and GA450 treatments were injected intraperitoneally with 0.5 mL of LPS (0.5 mg/kg body weight), while broilers in the CON group received an equal amount of normal saline.

Gallic acid (99.6% purity) was purchased from Wufeng Chicheng Biotechnology Co., Ltd. (Yichang, Hubei, China). LPS (*Escherichia coli* O55:B5) was purchased from Sigma-Aldrich Chemical Co., Ltd. (Saint Louis, MO, USA).

### 2.2. Diets and Chicken Husbandry

The basic diet (Table 1) was formulated based on the nutritional requirements of fast-growing yellow-feathered broilers in China’s “National Agricultural Industry Standard Nutrient Requirements for Yellow-feathered Broilers” (NY/T3645-2020) [13].

This experiment was conducted in the experimental area of the Institute of Animal Science of Guangdong Academy of Agricultural Sciences for 50 days. All experimental broilers were raised on the ground, with clean sawdust provided as bedding. The feeding management and environmental conditions of each treatment group were consistent. Broilers had free access to pellets and water and were vaccinated regularly. The room temperature was maintained at 27 to 30 °C. For the first 3 days, the light was kept on for 23 h a day, and then reduced by 2 h each day until it reached 16 h.

### 2.3. Measurements of Growth Performance 

The body weights of broiler chickens were measured at 1 day old and 50 days old, and daily feed intake was recorded. During the trial period, the health and mental state of the broiler chickens were observed daily. Mortality was checked daily, and any deceased chickens were weighed and recorded to appropriately adjust the estimated values for weight gain, intake, and feed conversion ratio. The final body weight (FBW), average daily feed intake (ADFI), average daily gain (ADG), feed/gain (F/G) ratio, and mortality were calculated. 

### 2.4. Sample Collection 

On the 50th day of the experiment, two broiler chickens with weights close to the average were selected from each replicate within each treatment for slaughter. The left pectoral muscle was taken to measure the color of the pectoral muscle, pH value, drip loss, shear force, and other indicators. The right pectoral muscle was rapidly frozen in liquid nitrogen and then stored at −80 °C for the detection of its antioxidant indices, shelf life indices, and related gene expressions. 

### 2.5. Determination of Meat Quality 

The lightness (L*), redness (a*), and yellowness (b*) values of slaughtered chickens’ pectoral muscles were measured using a colorimeter (CR-410, Konica Minolta, Tokyo, Japan) after 45 min and 24 h at room temperature. Each sample was measured three times and the average value was calculated. The pH of the slaughtered chickens’ pectoral muscles was determined using a muscle pH meter (HI8242, Hanna Instruments, Padova, Italy) after 45 min and 24 h of standing at room temperature, with each sample measured three times and the average value taken. The drip loss was measured according to the procedure reported by Jin et al. [14]. The left pectoral muscle was heated in a constant-temperature water bath to an internal temperature of approximately 70 °C. After cooling to room temperature, it was trimmed into pieces measuring 2 × 1 × 0.5 cm in length, width, and height, respectively, along the direction of the muscle fibers. The shear force was measured using an instrument (Instron 4411, Instron, Norwood, MA, USA). Each meat sample was tested eight times, and the average value was calculated.

### 2.6. Determination of Intramuscular Fat and Total Volatile Base Nitrogen Contents

Approximately 20 g of pectoral muscle samples were weighed; the fascia was removed, minced, placed in a Petri dish, and freeze-dried at −80 °C for 48 h. We accurately took 2 g of the freeze-dried sample and sealed it in a filter bag, then extracted it through a Soxhlet extraction device (XT15i, Ankom Technology, Macedon, NY, USA). After extraction, the filter paper tube was removed and the fat bottle was placed back into a constant-temperature oven at 105 °C for 3.5 h, then removed, cooled, and weighed to calculate the intramuscular fat (IMF) content. The total volatile base nitrogen (TVB-N) values were measured according to the method proposed by Chen et al. [15], with the results expressed as milligrams of TVB-N per 100 g of sample.

### 2.7. Determination of Pectoral Muscle Fatty Acid Composition

Approximately 1 g of pectoral muscle samples were weighed and homogenized. Then, 2 mL of hexane was added and the mixture was shaken at 50 °C for 30 min. Subsequently, 3 mL of KOH methanol solution (0.4 mol/L) was added and the mixture was shaken at 50 °C for an additional 30 min to derivatize. After that, an additional 1 mL of water and 2 mL of hexane were added, and the mixture was mixed well. The layers were allowed to separate and the upper layer was taken. The fatty acid composition and ratios were determined using a gas chromatograph (7890B-5977A, Agilent, Palo Alto, CA, USA).

### 2.8. Determination of Pectoral Muscle Antioxidant Index

Approximately 1 g of pectoral muscle sample was weighed and added to physiological saline at a ratio of 1:9 (sample: physiological saline) to prepare a 10% concentration homogenate. Then, commercially available total superoxide dismutase (T-SOD, A001-1-2), glutathione peroxidase (GSH-Px, A005-1-2), catalase (CAT, A007-1-1), total antioxidant capacity (T-AOC, A015-1-2), and malondialdehyde (MDA, A003-1-2) content assay kits were used to determine the MDA concentration and antioxidant enzyme activities in the muscle according to the manufacturer’s instructions (Nanjing Jiancheng Biological Technology Co., Ltd., Nanjing, China).

### 2.9. Expression of Related Genes in Pectoral Muscle

Total RNA from pectoral muscles was extracted using RNAiso plus (9109, TAKARA, Tokyo, Japan). Then, the PrimeScript II 1st Strand cDNA Synthesis Kit (6210A, TAKARA, Tokyo, Japan) was used to reverse-transcribe the extracted total RNA into cDNA. The real-time PCR reaction system was configured with broiler breast muscle cDNA as a template, with the primers from Table 2, and following the instructions of SYBR PremixEx Taq II (RR820A, TAKARA, Tokyo, Japan). The PCR reaction was carried out using a real-time fluorescence quantitative instrument (ABI7500, Applied Biosystems, Carlsbad, CA, USA). The relative expression of each target gene was determined using β-actin as an internal reference and the 2^–△△CT^ method.

### 2.10. Statistical Analysis

Effects of treatment were analyzed by the one-way analysis of variance (ANOVA) procedure in SPSS 22.0 (SPSS Inc., Chicago, IL, USA). Orthogonal polynomial contrasts were used to estimate the linear and quadratic effects of the increasing dietary GAs levels. Duncan’s multiple range tests were used to compare the treatment means, with significance values of *p* < 0.05. The tabulated results are shown as means with standard error of the mean (SEM).

## 3. Results

### 3.1. Growth Performance

As presented in Table 3, dietary supplementation with GA significantly increased (*p* < 0.05, linear *p* < 0.05) the FBW of broiler chickens. Compared to the LPS group, supplementation with 150 mg/kg GA significantly increased (*p* < 0.05) ADG and tended (*p* = 0.078) to increase ADFI. Different levels of GA had no significant effect (*p* > 0.05) on the Mortality and F/G of broiler chickens.

### 3.2. Meat Quality 

As presented in Table 4, compared to the CON group, the addition of 450 mg/kg GA significantly reduced (*p* < 0.05) the pH_45min_ value of broiler pectoral muscles. Compared with other groups, the addition of 450 mg/kg GA significantly increased the L*_45min_ value of pectoral muscles post-slaughter (*p* < 0.05) and significantly reduced (*p* < 0.05, linear *p* < 0.05) the drip loss value. Different levels of GA had no significant effect (*p* > 0.05) on the pH_24h_, L*_24h_, a*_24h_, b*_24h_, and shear force values of the pectoral muscle.

### 3.3. Intramuscular Fat and Total Volatile Basic Nitrogen Contents

As presented in Table 5, different GA levels had no significant effect (*p* > 0.05) on the IMF content of pectoral muscles. Compared with the CON group, adding 450 mg/kg GA significantly reduced (*p* < 0.05, linear *p* < 0.05) the TVB-N content in postmortem pectoral muscles.

### 3.4. Muscle Fatty Acid Composition

As presented in Table 6, the addition of GA had a trend effect (*p* = 0.063) on the content of C16:0 in the pectoral muscles of broilers after slaughter. Adding 450 mg/kg GA significantly increased (*p* < 0.05, linear *p* < 0.05) C22:6n-3 (docosahexaenoic acid, DHA) concentration in pectoral muscles. Different GA levels did not affect (*p* > 0.05) other fatty acids, total saturated fatty acids (SFA), total polyunsaturated fatty acids (PUFA), and the n-3/n-6 unsaturated fatty acid ratio in muscles.

### 3.5. Muscle Antioxidative Enzyme Activities and MDA Content

As presented in Table 7, the level of GA supplementation significantly affected (*p* < 0.05) T-SOD, CAT, GSH-Px activities and MDA content in pectoral muscles after slaughter. Compared with the other groups, supplementation with 150 mg/kg GA significantly increased (*p* < 0.05, linear or quadratic *p* < 0.05) T-SOD activity and significantly increased (*p* < 0.05, quadratic *p* < 0.05) CAT activity in pectoral muscles, and supplementation with 450 mg/kg GA significantly decreased (*p* < 0.05, linear *p* < 0.05) MDA content. Compared with the CON and LPS groups, supplementation with 150, 300, and 450 mg/kg GA had a tendency (*p* = 0.066, linear *p* < 0.05) to increase GSH-Px activity. Neither LPS stimulation nor GA supplementation had a significant effect on T-AOC level (*p* > 0.05).

### 3.6. Muscle MyHC mRNA Expression

**Figure 1:**
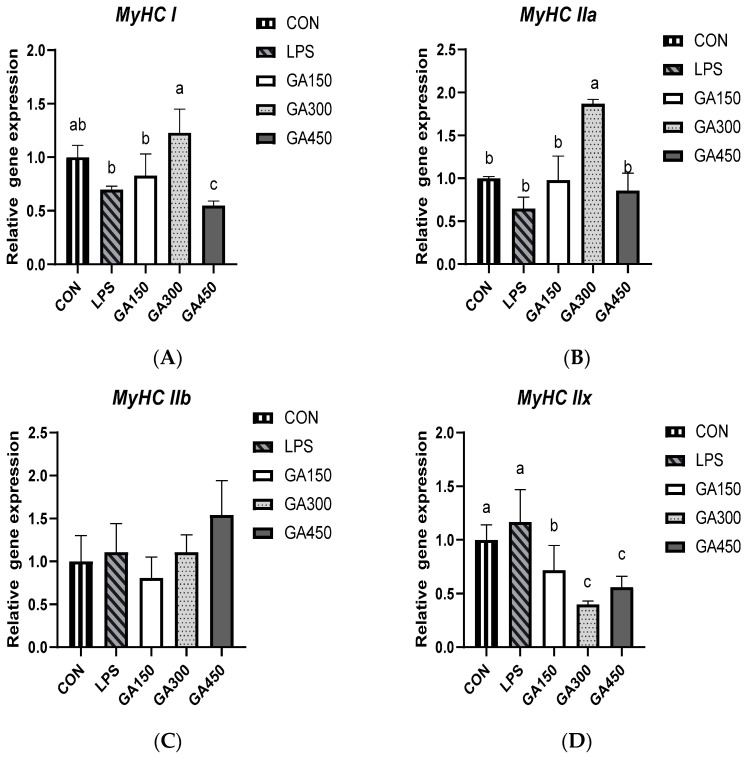
Effects of dietary GA on the mRNA expression of *MyHC I* (**A**), *MyHC IIa* (**B**), *MyHC IIx* (**C**), and *MyHC IIx* (**D**) in pectoral muscle. Data are means ± SEM of six replicates per treatment (25 birds per replicate). CON: basal diet and saline injection; LPS: basal diet and LPS challenge; GA150: basal diet supplemented with 150 mg/kg gallic acid and LPS challenge; GA300: basal diet supplemented with 300 mg/kg gallic acid and LPS challenge; GA450: basal diet supplemented with 450 mg/kg gallic acid and LPS challenge. Bars labeled with different letters significantly differ (*p* < 0.05).

As presented in Figure 1, the level of GA addition significantly affected (*p* < 0.05) the gene expression of the myosin heavy-chain genes *MyHC I*, *MyHC IIa* and *MyHC II*x in broiler pectoral muscles after slaughter. The addition of 300 mg/kg GA significantly increased (*p* < 0.05) the expression of *MyHC I* and *MyHC IIa* in broiler pectoral muscles compared to the LPS group (Figure 1A,B). Neither LPS stimulation nor the addition of GA had a significant effect on the expression of *MyHC IIb* (*p* > 0.05; Figure 1C). The addition of 150, 300, and 450 mg/kg GA significantly decreased (*p* < 0.05) the expression of *MyHC IIx* compared to the LPS group (Figure 1D).

## 4. Discussion

Growth performance is one of the most important indicators to evaluate whether an additive is effective or not. LPS stimulation, as a stress model, can change the content of water and electrolytes in the small intestine, causing an imbalance of homeostasis in the intestinal tract of broiler chickens, and can reduce the growth performance of poultry [16]. Zhang et al. injected LPS into the peritoneal cavity of AA chickens on days 16, 18, and 20, which significantly reduced the body weight gain of broilers at the ages of 1 to 21 days [17]. This is similar to the results of the present experiment. Therefore, alleviating the decline in growth performance caused by LPS stimulation could also reflect the effect of the additive. Samuel et al. increased feed conversion ratios from 1 to 42 days of age by adding 75–100 mg/kg of GA to 1-day-old AA broiler diets [18]. This was similar to the results of the present experiment, where GA addition increased body weight and decreased FCR in the starter stage, finishing stage, and whole stage compared to the LPS-stimulated group. Another study showed that the addition of grape pomace rich in GA to drinking water increased the body weight of broiler chickens by 5.8% and resulted in a cecal microbial composition that was more favorable for energy deposition in broiler chickens, thereby promoting weight gain in broiler chickens [19]. In addition, GA has been shown to have antimicrobial properties, induce changes in the microbiota toward a more favorable composition and activity, promote gastrointestinal epithelial cell proliferation and differentiation, and repair damaged mucosa to improve nutrient digestion and absorption [20,21,22]. Therefore, the positive effects on growth performance shown in this study can be explained by the fact that GA may counteract the impairment of LPS on the digestion and absorption capacity of broiler chickens by suppressing the inflammatory response and targeting the intestine while maintaining its barrier function.

Muscle pH value is an important index to evaluate muscle quality and the decomposition rate of muscle glycogen after poultry slaughter, which is mainly affected by phosphofructokinase activity [23]. The amount of lactic acid produced in muscle is related to the glycogen content in the muscle. The more glycogen reserves in the muscle, the more lactic acid is produced during strenuous exercise, the more it accumulates, and the lower the pH value, which leads to meat acidification. In addition, studies have shown that the pH value of pectoral muscles is negatively correlated with lightness, yellowness, and shear force [24], and differences in muscle fiber composition, density, and diameter can affect muscle pH [25]. In the present study, the addition of 150 mg/kg GA reduced the pH value of pectoral muscles 45 min after slaughter. It has been reported that GA added to the diet can be distributed to tissues throughout the body [26], which could be responsible for the decrease in the pH value of muscle tissue. The meat color is one of the main quality attributes that directly influence consumer choice. Fresh chicken breasts are slightly pink, but may also appear white to yellow due to several factors [27]. It has been reported that the color of meat may be influenced by the concentration of myoglobin, its chemical and physical state, and the structure of the meat surface [27,28]. Drip loss is a major indicator of muscle water-holding capacity. Muscle with weak water-holding capacity has accelerated loss of total pigments and soluble nutrients, resulting in impaired meat color and flavor [29]. Muscle lightness was correlated with drip loss, with higher lightness being associated with poorer water-holding capacity and relatively higher drip loss. The results of the present study showed that the addition of 450 mg/kg GA led to a higher pectoral muscle lightness value compared to the LPS and CON groups. Increased lightness value and drip loss often imply accelerated muscle protein degradation [30], and lower pH reduces the hydrolytic potential of proteins, which reduces the muscle’s water-locking capacity, and free water in the muscle seeps out of the surface of the muscle, which leads to an increase in lightness [31].

Oxidative reactions are still going on in the muscle after the animal is slaughtered. The activities of auto-antioxidant enzymes decrease continuously with the prolongation of post-slaughter muscle placement time, and if they decrease to a level that does not effectively reduce ROS, it can lead to muscle quality degradation and the production of toxic and hazardous substances, such as volatile saline nitrogen, thus affecting the shelf life of the product, which is not conducive to the health of the consumer [32]. The volatile basic nitrogen content, as an indicator of the proteolysis level, increased with storage time, thus accelerating muscle spoilage [33]. The addition of 450 mg/kg GA significantly reduced the volatile basic nitrogen content of the pectoral muscle. In addition to its antioxidant function, this may be due to the fact that GA inhibited the growth of *Pseudomonas* spp. and *Enterobacter* spp. in muscle, which can use amino acids as a growth substrate to produce sulfur-containing compounds and amines with an off-flavor, resulting in a rapid increase in the volatile basic nitrogen content [34,35]. Our test results suggest that the addition of GA is beneficial to improving the storage stability of muscles and prolong their shelf life.

Eating foods rich in n-3 polyunsaturated fatty acids (n-3 PUFA) and essential fatty acids can play a natural preventive role in cardiovascular disease and other health problems [36,37]. The nutritional quality of chicken is an important factor affecting the health of consumers. Studies have found that PUFA can be effectively deposited in chicken and improve the quality of chicken meat [38]. The fatty acid composition of intramuscular fat, on the other hand, has implications for human health, and it is generally recognized that higher PUFA/SFA ratios are less likely to increase the incidence of cardiovascular disease and that PUFA/SFA > 0.45 barely increases the incidence of cardiovascular disease [39]. However, lipid oxidation increases linearly with increasing PUFA content, and the oxidative stability of unsaturated fatty acids (UFA) decreases with increasing levels of unsaturation. Omega-3 long-chain unsaturated fatty acids, especially docosahexaenoic acid (DHA, C22:6n-3), are very sensitive to oxidation, and the body’s antioxidant capacity has a significant effect on its content [40]. DHA can promote brain and retinal development, inhibit inflammation, and reduce the risk of cardiovascular disease and obesity [41]. Studies have shown that adding basil, thyme, and sage to the diet can significantly increase healthy ALA (C18:3n-3), EPA (C20:5n-3), DHA, and total PUFA content and improve the fatty acid profile in broiler muscles [42]. In this experiment, the addition of GA not only improved the antioxidant properties of broiler thigh muscle, but also enhanced its nutritional value by increasing health-promoting n-3 PUFA. Since there was no change in unsaturated fatty acid content in the diets of each stage, the higher unsaturated fatty acid content in the thigh meat of broilers supplemented with natural feed additives may be due to the protective effect of antioxidants on the oxidative decomposition of unsaturated fatty acids. Therefore, GA may increase the relative content of DHA in pectoral muscles through antioxidant action, thus improving the nutritional quality of muscles.

Under physiological conditions, animals produce reactive oxygen species (ROS), which can cause damage to the body [43]. Multiple studies have shown that polyphenols can improve tissue oxidative status by scavenging free radicals and increasing the activity of antioxidant enzymes [44,45,46]. The dietary addition of plant polysaccharides, polyphenols, and other bioactive substances can improve the antioxidant capacity of muscles and improve muscle quality [47,48]. GA is a good natural antioxidant that can eliminate ROS such as superoxide anions, hydrogen peroxide, and hydroxyl radicals [49,50]. Ramay et al. have shown that antioxidant enzymes such as CAT, SOD, and GSH-Px can work together to reduce MDA content, remove excess ROS, reduce oxidative damage to pectoral muscles, and thus maintain better muscle mass [47]. In this experiment, the addition of GA to the diet alleviated the effects of an LPS-induced increase in muscle MDA content and a decrease in T-SOD, GSH-PX, and CAT activities. This is consistent with previous studies in which it was found that GA can improve the antioxidant capacity of broiler chicken muscles [11,18]. In summary, our results further confirmed that GA can effectively exert its antioxidant function to ensure animal health and growth.

Shear force is an indicator of muscle tenderness, which is inversely related to tenderness, and is related to myofiber diameter and fascicle membrane thickness. The myosin heavy-chain method of classifying muscle fiber types is based on their ATPase activity and maximum contraction velocity, classifying them into four different major isoforms: slow-oxidizing (I), fast-oxidizing (IIa), fast-fermenting (IIb), and intermediate (IIx) [51]. Aerobic endurance exercise in humans and animals effectively induces skeletal muscle type remodeling, i.e., the gradual transformation of skeletal muscle fibers from fast muscle (high proportion of *MyHC* type IIb fibers) to slow muscle (high proportion of *MyHC* type I fibers) [52]. This adaptive remodeling process of muscle fiber type is crucial for the body’s energy homeostasis and fatigue alleviation, and the improvement of the meat quality of livestock and poultry [53]. Type I fibers contain more mitochondria and aerobic metabolic enzymes such as cytochrome oxidase and succinate dehydrogenase, while type IIb fibers contain fewer mitochondria and more glycogen and glycolytic enzymes, and type IIa fibers are involved in glycolysis and aerobic oxidation. The heme content of type IIb fibers was lower than that of other types of fibers, the proportion of oxidized myosin heavy chain in muscles negatively correlated with myofibril diameter, and the proportion of the intermediate type positively correlated with the proportion of myofibrils [54]. This experiment found that the addition of GA increased the gene expression of oxidized myosin heavy chain in the pectoral muscle of broiler chickens, reduced the gene expression of intermediate myosin heavy chain, promoted the differentiation of muscle fibers from heavy-chain types to oxidized muscle fibers, reduced the diameter of pectoral muscle fibers, and increased their density. This suggests that GA may reduce muscle shear force, increase muscle tenderness, and improve meat quality by regulating the muscle fiber structure type. This is similar to the findings on polyphenol in fattening pigs [55].

## 5. Conclusions

In conclusion, the dietary addition of GA may alleviate the negative impact of stress response on the growth performance of broiler chickens and enhance their antioxidant capacity. It also promoted the differentiation of the heavy-chain type of glycolytic myofibrils to oxidized myofibrils, which effectively improved the quality of broiler pectoral muscle. The appropriate amount of dietary GA at each stage was 300 mg/kg.

## Figures and Tables

**Table 1:** Composition and nutrient levels of basal diet (air-dry basis, %).

Items	1 to 21 d	22 to 42 d	43 to 50 d
Ingredients, %			
Corn	52.07	51.80	51.00
Wheat	5.00	10.00	15.00
Soybean meal	33.00	25.80	20.60
Corn gluten meal	3.00	4.50	5.00
Soybean oil	2.28	3.50	4.40
*L*-Lysine HCl	0.25	0.24	0.20
*DL*-Methionine	0.17	0.14	0.08
Limestone	1.25	1.16	1.10
CaHPO_4_	1.68	1.56	1.32
NaCl	0.30	0.30	0.30
Premix ^1^	1.00	1.00	1.00
Total	100.00	100.00	100.00
Nutritional levels ^2^			
ME/(Kcal/kg)	2960	3060	3155
CP %	23.11	21.40	19.38
Ca %	1.33	1.21	1.19
Total phosphorus %	0.76	0.7	0.71
Nonphytate phosphorus P %	0.47	0.41	0.36
Lysine %	1.29	1.15	0.96
Methionine %	0.52	0.48	0.40
Threonine %	0.86	0.81	0.67

^1^ Premix supplemented with the following per kg of the diet (1 to 21 d of age): Vit. A, 12000 IU; Vit. D_3_, 600 IU; Vit. E, 45 IU; Vit. K_3_, 2.5 mg; Vit. B_1_, 2.4 mg; Vit. B_2_, 5 mg; Vit. B_6_, 2.8 mg; Vit. B_12_, 16 μg; pantothenic acid (P.A.), 12 mg; niacin, 42 mg; biotin, 0.12 mg; choline chloride, 1300 mg; Fe, 80 mg; Cu, 7 mg; Mn, 80 mg; Zn, 85 mg; Se, 0.15 mg; I, 0.7 mg. Premix added into the diet per kg (22 to 42 d of age): Vit. A, 9000 IU; Vit. D_3_, 600 IU; Vit.E, 45 IU; Vit. K_3_, 2.5 mg; Vit. B_1_, 2.4 mg; Vit. B_2_, 5.0 mg; Vit. B_6_, 2.8 mg; Vit. B_12_, 16 μg; pantothenic acid (P.A.) 12 mg; niacin, 42 mg; biotin, 0.12 mg; choline chloride, 1000 mg; Fe, 80 mg; Cu, 7 mg; Mn, 80 mg; Zn, 80 mg; Se, 0.15 mg; I, 0.7 mg. Premix added into the diet per kg (43 to 50 d of age): Vit. A, 6000 IU; Vit. D_3_, 500 IU; Vit.E, 25 IU; Vit. K_3_, 1.7 mg; Vit. B_1_, 1.0 mg; Vit. B_2_, 4.0 mg; Vit. B_6_, 0.6 mg; Vit. B_12_, 8.0 μg; pantothenic acid (P.A.), 8 mg; niacin, 20 mg; biotin, 0.02 mg; choline chloride, 750 mg; Fe, 80 mg; Cu, 7 mg; Mn, 80 mg; Zn, 75 mg; Se, 0.15 mg; I, 0.7 mg. ^2^ CP, Ca, and total phosphorus were measured values, while the others were calculated.

**Table 2:** Primers for real-time PCR of pectoral muscles.

Gene ^1^	GenBank ID	Primers Sequence (5′-3′)	Tm, ℃
*β*-actin	NM_001001611.2	F: GAGAAATTGTGCGTGACATCA	56
		R: CCTGAACCTCTCATTGCCA	
*MyHC I*	NM_204587.2	F: ACGGGCCTGATCAACCAAAA	56
		R: GGCCTTCTTGGCTTTCTCCT	
*MyHC IIa*	NM_204228.3	F: GGTCAACAAGCTCCGAGTGA	56
		R: CAGGCCACTTTACTGCCTCA	
*MyHC IIb*	NM_001013396.1	F: GGGAGACCTGAATGAAATGGAG	56
		R: CTTCCTGTGACCTGAGAGCATC	
*MyHC IIx*	NC_052549.1	F: AGACACATTTGCTGCACTGG	56
		R: GGTCCGTGCCTTTCAGTTTT	

^1^ *MyHC* = myosin heavy chain.

**Table 3:** Effect of dietary GA supplementation on growth performance in broiler chickens ^1^.

	Treatments ^3^					SEM ^4^	*p*-Values ^5^		
Items ^2^	CON	LPS	GA150	GA300	GA450		ANOVA	Linear	Quadratic
IBW (g)	39.41	39.55	39.53	39.44	39.52	0.05	0.526	0.801	0.670
FBW (kg)	1.69 ^b^	1.71 ^b^	1.78 ^a^	1.75 ^ab^	1.75 ^ab^	0.02	0.031	0.019	0.086
ADFI (g/d)	65.17	65.98	66.78	65.41	66.40	0.35	0.078	0.340	0.175
ADG (g/d)	33.17 ^b^	33.98 ^b^	34.77 ^a^	33.84 ^b^	34.54 ^a^	0.20	0.039	0.072	0.056
F/G (g/g)	1.96	1.94	1.92	1.93	1.92	0.01	0.278	0.093	0.437
Mortality (%)	4.00	9.33	7.33	5.33	9.34	2.57	0.466	0.572	0.359

^1^ Data are means of six replicates per treatment (25 birds per replicate). Values with various superscript letters in same row are significantly different (*p* < 0.05). ^2^ IBW = initial body weight; FBW = final body weight; ADFI = average daily feed intake; ADG = average daily gain; F/G = ratio of feed to gain. ^3^ CON: basal diet and saline injection; LPS: basal diet and LPS challenge; GA150: basal diet supplemented with 150 mg/kg gallic acid and LPS challenge; GA300: basal diet supplemented with 300 mg/kg gallic acid and LPS challenge; GA450: basal diet supplemented with 450 mg/kg gallic acid and LPS challenge. ^4^ SEM: standard error of mean. ^5^ ANOVA: treatment effect according to Duncan’s test; Linear and Quadratic: linear and quadratic dose trend effect of GA at 0, 150, 300, and 450 mg/kg according to polynomial contrasts.

**Table 4:** Effect of dietary GA supplementation on meat quality of broilers ^1^.

	Treatments ^3^					SEM ^4^	*p*-Values ^5^		
Items ^2^	CON	LPS	GA150	GA300	GA450		ANOVA	Linear	Quadratic
pH_45min_	5.98 ^a^	5.91 ^ab^	5.97 ^a^	5.86 ^ab^	5.85 ^b^	0.02	0.035	0.554	0.070
pH_24h_	5.61	5.74	5.68	5.58	5.67	0.01	0.234	0.784	0.369
L*_45min_	57.74 ^bc^	56.86 ^c^	58.47 ^ab^	58.27 ^ab^	59.56 ^a^	0.47	0.008	0.144	0.163
a*_45min_	13.28	13.50	13.25	13.17	11.90	0.16	0.163	0.469	0.451
b*_45min_	18.00	17.03	17.28	17.42	17.93	0.23	0.130	0.947	0.319
L*_24h_	62.84	61.62	61.84	61.40	62.65	0.89	0.726	0.834	0.211
a*_24h_	11.67	12.20	12.98	13.00	11.80	0.16	0.269	0.415	0.125
b*_24h_	19.18	18.11	18.31	18.17	21.18	0.26	0.072	0.121	0.143
Shear force, N	28.77	31.19	26.42	26.01	17.21	0.90	0.109	0.084	0.202
Drip loss, %	2.28 ^b^	2.87 ^a^	2.41 ^b^	2.42 ^b^	2.27 ^b^	0.07	0.051	0.317	0.085

^1^ Data are means of six replicates per treatment (25 birds per replicate). Values with various superscript letters in same row are significantly different (*p* < 0.05). ^2^ pH_45min_ = pH at 45 min postmortem; pH_24h_ = pH at 24 h postmortem; meat color (L*, a*, and b* values), shear force, and drip loss were measured at 45 min and 24 h postmortem. ^3^ CON: basal diet and saline injection; LPS: basal diet and LPS challenge; GA150: basal diet supplemented with 150 mg/kg gallic acid and LPS challenge; GA300: basal diet supplemented with 300 mg/kg gallic acid and LPS challenge; GA450: basal diet supplemented with 450 mg/kg gallic acid and LPS challenge. ^4^ SEM: standard error of mean. ^5^ ANOVA: treatment effect according to Duncan’s test; Linear and Quadratic: linear and quadratic dose trend effect of GA at 0, 150, 300, and 450 mg/kg according to polynomial contrasts.

**Table 5:** Effect of dietary EA supplementation on IMF and TVB-N contents of pectoral muscles in broilers ^1^.

	Treatments ^3^					SEM ^4^	*p*-Values ^5^		
Items ^2^	CON	LPS	GA150	GA300	GA450		ANOVA	Linear	Quadratic
IMF, %	1.54	1.26	1.30	1.44	1.60	0.01	0.509	0.481	0.359
TVB-N, mg/100 g	12.72 ^a^	12.09 ^ab^	12.43 ^ab^	12.08 ^ab^	11.64 ^b^	0.14	0.007	0.023	0.789

^1^ Data are means of six replicates per treatment (25 birds per replicate). Values with various superscript letters in same row are significantly different (*p* < 0.05). ^2^ IMF = intramuscular fat; TVB-N = total volatile basic nitrogen. ^3^ CON: basal diet and saline injection; LPS: basal diet and LPS challenge; GA150: basal diet supplemented with 150 mg/kg gallic acid and LPS challenge; GA300: basal diet supplemented with 300 mg/kg gallic acid and LPS challenge; GA450: basal diet supplemented with 450 mg/kg gallic acid and LPS challenge. ^4^ SEM: standard error of mean. ^5^ ANOVA: treatment effect according to Duncan’s test; Linear and Quadratic: linear and quadratic dose trend effect of GA at 0, 150, 300, and 450 mg/kg according to polynomial contrasts.

**Table 6:** Effect of dietary GA supplementation on fatty acid composition of pectoral muscles in broilers (%) ^1^.

	Treatments ^3^					SEM ^4^	*p*-Values ^5^		
Items ^2^	CON	LPS	GA150	GA300	GA450		ANOVA	Linear	Quadratic
C14:0	0.48	0.50	0.48	0.48	0.45	0.02	0.274	0.253	0.103
C16:0	21.57	22.57	21.82	21.42	20.85	0.22	0.063	0.094	0.125
C18:0	8.51	8.92	8.39	8.39	8.33	0.39	0.821	0.346	0.472
C20:0	0.22	0.23	0.22	0.22	0.21	0.01	0.844	0.921	0.447
C22:0	0.72	0.73	0.69	0.80	0.65	0.08	0.741	0.250	0.533
Total SFA	32.26	32.78	32.01	31.93	31.01	0.80	0.138	0.355	0.202
C16:1	2.86	3.25	3.20	2.62	2.52	0.28	0.248	0.358	0.821
C18:1	26.72	26.9	27.13	25.95	25.17	0.84	0.462	0.760	0.879
Total MUFA	29.58	30.15	30.33	28.57	27.68	1.00	0.319	0.612	0.948
C18:2n-6	28.32	27.87	29.35	29.65	29.35	0.97	0.663	0.443	0.298
C18:3n-3	0.29	0.29	0.30	0.30	0.31	0.01	0.920	0.915	0.688
C20:2n-6	0.59	0.54	0.60	0.66	0.59	0.05	0.578	0.499	0.163
C20:3n-6	4.02	3.88	4.03	4.34	5.00	0.47	0.578	0.324	0.309
C20:5n-3	0.93	0.92	0.93	0.98	1.13	0.12	0.724	0.237	0.545
C22:6n-3	0.48 ^b^	0.49 ^b^	0.51 ^ab^	0.54 ^ab^	0.68 ^a^	0.07	0.019	0.041	0.150
Total PUFA	34.64	34.09	36.01	36.04	36.97	0.86	0.155	0.984	0.059
n-6	32.88	32.33	34.28	34.28	34.86	0.84	0.201	0.767	0.071
n-3	1.73	1.77	1.77	1.86	2.11	0.18	0.486	0.115	0.401
n-3/n-6	0.05	0.05	0.05	0.06	0.06	0.00	0.667	0.099	0.746

^1^ Data are means of six replicates per treatment (25 birds per replicate). Values with various superscript letters in same row are significantly different (*p* < 0.05). ^2^ SFA = saturated fatty acids; MUFA = monounsaturated fatty acids; PUFA = polyunsaturated fatty acids. ^3^ CON: basal diet and saline injection; LPS: basal diet and LPS challenge; GA150: basal diet supplemented with 150 mg/kg gallic acid and LPS challenge; GA300: basal diet supplemented with 300 mg/kg gallic acid and LPS challenge; GA450: basal diet supplemented with 450 mg/kg gallic acid and LPS challenge. ^4^ SEM: standard error of mean. ^5^ ANOVA: treatment effect according to Duncan’s test; Linear and Quadratic: linear and quadratic dose trend effect of GA at 0, 150, 300, and 450 mg/kg according to polynomial contrasts.

**Table 7:** Effect of dietary GA supplementation on antioxidant capacity of pectoral muscles in broilers ^1^.

	Treatments ^3^					SEM ^4^	*p*-Values ^5^		
Items ^2^	CON	LPS	GA150	GA300	GA450		ANOVA	Linear	Quadratic
T-SOD, U/mg prot	301.41 ^c^	318.84 ^bc^	362.90 ^a^	357.21 ^a^	339.63 ^ab^	2.05	0.001	0.045	0.019
GSH-Px, U/mg prot	31.18	37.75	43.99	44.28	43.77	3.37	0.066	0.022	0.254
CAT, U/mg prot	32.55 ^bc^	30.30 ^c^	39.63 ^a^	37.62 ^ab^	32.89 ^bc^	9.79	0.013	0.208	0.035
T-AOC, U/mg prot	1.31	1.27	1.31	1.25	1.27	0.01	0.242	0.091	0.816
MDA, nmol/mg prot	2.41 ^a^	2.45 ^a^	1.76 ^ab^	1.59 ^b^	1.54 ^b^	0.01	0.038	0.004	0.641

^1^ Data are means of six replicates per treatment (25 birds per replicate). Values with various superscript letters in same row are significantly different (*p* < 0.05). ^2^ T-SOD = total superoxide dismutase; GSH-Px = glutathione peroxidase; CAT = catalase; T-AOC = total antioxidative capacity; MDA = malonic dialdehyde. ^3^ CON: basal diet and saline injection; LPS: basal diet and LPS challenge; GA150: basal diet supplemented with 150 mg/kg gallic acid and LPS challenge; GA300: basal diet supplemented with 300 mg/kg gallic acid and LPS challenge; GA450: basal diet supplemented with 450 mg/kg gallic acid and LPS challenge. ^4^ SEM: standard error of mean. ^5^ ANOVA: treatment effect according to Duncan’s test; Linear and Quadratic: linear and quadratic dose trend effect of GA at 0, 150, 300 and 450 mg/kg according to polynomial contrasts.

## Data Availability

All datasets collected and analyzed during the current study are available from the corresponding author by request; the availability of the data is restricted to investigators based at academic institutions.

## References

[1] Wierup M. (2000). The control of microbial diseases in animals: Alternatives to the use of antibiotics. Int. J. Antimicrob. Agents.

[2] Casewell M., Friis C., Marco E., McMullin P., Phillips I. (2003). The European ban on growth-promoting antibiotics and emerging consequences for human and animal health. J. Antimicrob. Chemother..

[3] Chen Y., Zhang H., Cheng Y., Li Y., Wen C., Zhou Y. (2018). Dietary l-threonine supplementation attenuates lipopolysaccharide-induced inflammatory responses and intestinal barrier damage of broiler chickens at an early age. Br. J. Nutr..

[4] Raetz C.R., Whitfield C. (2002). Lipopolysaccharide endotoxins. Annu. Rev. Biochem..

[5] Huang Z., Jin S., Lv Z. (2022). Dietary genistein supplementation alters mRNA expression profile and alternative splicing signature in the thymus of chicks with lipopolysaccharide challenge. Poul. Sci..

[6] Zhong W.J., Yang H.H., Guan X.X., Xiong J.B., Sun C.C., Zhang C.Y., Luo X.Q., Zhang Y.F., Zhang J., Duan J.X. (2019). Inhibition of glycolysis alleviates lipopolysaccharide-induced acute lung injury in a mouse model. J. Cell. Physiol..

[7] Wang Y., Ye J., Zhang S., Chen Z., Fan Q., Jiang S. (2023). Dietary supplementation with anthocyanin attenuates lipopolysaccharide-induced intestinal damage through antioxidant effects in yellow-feathered broiler chicks. Poul. Sci..

[8] De Cesare A., Sirri F., Manfreda G., Moniaci P., Giardini A., Zampiga M., Meluzzi A. (2017). Effect of dietary supplementation with *Lactobacillus acidophilus* D2/CSL (CECT 4529) on caecum microbioma and productive performance in broiler chickens. PLoS ONE.

[9] Arshad R., Mohyuddin A., Saeed S., Hassan A.U. (2021). Optimized production of tannase and gallic acid from fruit seeds by solid state fermentation. Trop. J. Pharm. Res..

[10] Li Y., Xie Z., Gao T., Li L., Chen Y., Xiao D., Liu W., Zou B., Lu B., Tian X. (2019). A holistic view of gallic acid-induced attenuation in colitis based on microbiome-metabolomics analysis. Food. Funct..

[11] Starčević K., Krstulović L., Brozić D., Maurić M., Stojević Z., Mikulec Ž., Bajić M., Mašek T. (2015). Production performance, meat composition and oxidative susceptibility in broiler chicken fed with different phenolic compounds. J. Sci. Food. Agric..

[12] Lee K.H., Jung S., Kim H.J., Kim I.S., Lee J.H., Jo C. (2012). Effect of dietary supplementation of the combination of gallic and linoleic acid in thigh meat of broilers. Asian-Australas. J. Anim. Sci..

[13] (2020). Nutrient Requirements for Yellow-Feathered Broilers.

[14] Jin C.-L., Wang Q., Zhang Z.-M., Xu Y.-L., Yan H.-C., Li H.-C., Gao C.-Q., Wang X.-Q. (2018). Dietary supplementation with pioglitazone hydrochloride and chromium methionine improves growth performance, meat quality, and antioxidant ability in finishing pigs. J. Agric. Food. Chem..

[15] Chen J., Wang S.Z., Chen J.Y., Chen D.Z., Deng S.G., Xu B. (2019). Effect of cold plasma on maintaining the quality of chub mackerel (Scomber japonicus): Biochemical and sensory attributes. J. Sci. Food. Agric..

[16] He Z., Li Y., Xiong T., Nie X., Zhang H., Zhu C. (2022). Effect of dietary resveratrol supplementation on growth performance, antioxidant capacity, intestinal immunity and gut microbiota in yellow-feathered broilers challenged with lipopolysaccharide. Front. Microbiol..

[17] Zhang L.-Z., Gong J.-G., Li J.-H., Hao Y.-S., Xu H.-J., Liu Y.-C., Feng Z.-H. (2023). Dietary resveratrol supplementation on growth performance, immune function and intestinal barrier function in broilers challenged with lipopolysaccharide. Poult. Sci..

[18] Samuel K., Wang J., Yue H., Wu S., Zhang H., Duan Z., Qi G. (2017). Effects of dietary gallic acid supplementation on performance, antioxidant status, and jejunum intestinal morphology in broiler chicks. Poult. Sci..

[19] Salaheen S., Kim S.-W., Haley B.J., Van Kessel J.A.S., Biswas D. (2017). Alternative growth promoters modulate broiler gut microbiome and enhance body weight gain. Front. Microbiol..

[20] Borges A., Ferreira C., Saavedra M.J., Simões M. (2013). Antibacterial activity and mode of action of ferulic and gallic acids against pathogenic bacteria. Microb. Drug. Resist..

[21] Pandurangan A.K., Mohebali N., Esa N.M., Looi C.Y., Ismail S., Saadatdoust Z. (2015). Gallic acid suppresses inflammation in dextran sodium sulfate-induced colitis in mice: Possible mechanisms. Int. Immunopharmacol..

[22] Dhingra M.S., Dhingra S., Chadha R., Singh T., Karan M. (2014). Design, synthesis, physicochemical, and pharmacological evaluation of gallic acid esters as non-ulcerogenic and gastroprotective anti-inflammatory agents. Med. Chem. Res..

[23] Wang C., Matarneh S.K., Gerrard D., Tan J. (2021). Modelling of energy metabolism and analysis of pH variations in postmortem muscle. Meat. Sci..

[24] Frerichs C., Beaulac K., Crowe T., Schwean-Lardner K. (2021). The effects of simulated transport on the muscle characteristics of white-feathered end-of-cycle hens. Poult. Sci..

[25] Huo W., Weng K., Gu T., Zhang Y., Zhang Y., Chen G., Xu Q. (2021). Effect of muscle fiber characteristics on meat quality in fast-and slow-growing ducks. Poult. Sci..

[26] Yang K., Zhang L., Liao P., Xiao Z., Zhang F., Sindaye D., Xin Z., Tan C., Deng J., Yin Y. (2020). Impact of gallic acid on gut health: Focus on the gut microbiome, immune response, and mechanisms of action. Front. Immunol..

[27] Bennato F., Ianni A., Martino C., Grotta L., Martino G. (2021). Evaluation of chemical composition and meat quality of breast muscle in broilers reared under light-emitting diode. Animals.

[28] Lindahl G., Lundström K., Tornberg E. (2001). Contribution of pigment content, myoglobin forms and internal reflectance to the colour of pork loin and ham from pure breed pigs. Meat. Sci..

[29] Gao Z., Yin J., Zhang J., Ward R.E., Martin R.J., Lefevre M., Cefalu W.T., Ye J. (2009). Butyrate improves insulin sensitivity and increases energy expenditure in mice. Diabetes.

[30] Lan R., Zhao Z., Li S., An L. (2020). Sodium butyrate as an effective feed additive to improve performance, liver function, and meat quality in broilers under hot climatic conditions. Poult. Sci..

[31] Rey A., Menoyo D., Segura J., López-Bote C., Calvo L. (2020). Combination of dietary glycaemic index and fasting time prior to slaughter as strategy to modify quality of pork. Meat. Sci..

[32] Onk K., Yalcintan H., Sari M., Isik S.A., Yakan A., Ekiz B. (2019). Effects of genotype and sex on technological properties and fatty acid composition of duck meat. Poult. Sci..

[33] Kim H.-J., Shin D.-J., Kim H.-J., Cho J., Kwon J.-S., Kim D., Jung J.-H., Jang A. (2022). Assessment of chicken thigh meat quality of Ross 308 broiler of animal welfare certified farm. Anim. Biosci..

[34] Sujiwo J., Kim D., Jang A. (2018). Relation among quality traits of chicken breast meat during cold storage: Correlations between freshness traits and torrymeter values. Poult. Sci..

[35] Jung SamOoel J.S., Bae YoungSik B.Y., Kim HyunJoo K.H., Jayasena D., Lee JunHeon L.J., Park HeeBok P.H., Heo KangNyung H.K., Jo CheoRun J.C. (2013). Carnosine, anserine, creatine, and inosine 5′-monophosphate contents in breast and thigh meats from 5 lines of Korean native chicken. Poult. Sci..

[36] Ruxton C., Reed S.C., Simpson M., Millington K. (2007). The health benefits of omega-3 polyunsaturated fatty acids: A review of the evidence. J. Hum. Nutr. Diet..

[37] Riediger N.D., Othman R.A., Suh M., Moghadasian M.H. (2009). A systemic review of the roles of n-3 fatty acids in health and disease. J. Am. Diet. Assoc..

[38] Gou Z., Cui X., Li L., Fan Q., Lin X., Wang Y., Jiang Z., Jiang S. (2020). Effects of dietary incorporation of linseed oil with soybean isoflavone on fatty acid profiles and lipid metabolism-related gene expression in breast muscle of chickens. Animal.

[39] Burghardt P.R., Kemmerer E.S., Buck B.J., Osetek A.J., Yan C., Koch L.G., Britton S.L., Evans S.J. (2010). Dietary n-3: n-6 fatty acid ratios differentially influence hormonal signature in a rodent model of metabolic syndrome relative to healthy controls. Nutr. Metab..

[40] Vlaicu P.A., Panaite T.D., Turcu R.P. (2021). Enriching laying hens eggs by feeding diets with different fatty acid composition and antioxidants. Sci. Rep..

[41] Shen Y., Guo C., Lu T., Ding X.-Y., Zhao M.-T., Zhang M., Liu H.-L., Song L., Zhou D.-Y. (2021). Effects of gallic acid alkyl esters and their combinations with other antioxidants on oxidative stability of DHA algae oil. Food. Res. Int..

[42] Mourão J.L., Pinheiro V., Prates J., Bessa R., Ferreira L., Fontes C., Ponte P. (2008). Effect of dietary dehydrated pasture and citrus pulp on the performance and meat quality of broiler chickens. Poul. Sci..

[43] Favier A. (1997). Oxidative stress: Value of its demonstration in medical biology and problems posed by the choice of a marker. Ann. Biol. Clin..

[44] Du E., Fan Q., Zhao N., Zhang W., Wei J., Chen F., Huang S., Guo W. (2021). Supplemental magnolol improves the antioxidant capacity and intestinal health of broiler chickens. Anim. Sci. J..

[45] Lu S.-H., Chen T.-H., Chou T.-C. (2015). Magnolol Inhibits RANKL-induced osteoclast differentiation of raw 264.7 macrophages through heme oxygenase-1-dependent inhibition of NFATc1 expression. J. Nat. Prod..

[46] Nishiyama T., Masuda Y., Izawa T., Ohnuma T., Ogura K., Hiratsuka A. (2019). Magnolol protects PC12 cells from hydrogen peroxide or 6-hydroxydopamine induced cytotoxicity. J. Toxicol. Sci..

[47] Ramay M.S., Yalçın S. (2020). Effects of supplemental pine needles powder (*Pinus brutia*) on growth performance, breast meat composition, and antioxidant status in broilers fed linseed oil-based diets. Poult. Sci..

[48] Long L., Zhang H., Wang F., Yin Y., Yang L., Chen J. (2021). Research Note: Effects of polysaccharide-enriched Acanthopanax senticosus extract on growth performance, immune function, antioxidation, and ileal microbial populations in broiler chickens. Poult. Sci..

[49] Yen G.-C., Duh P.-D., Tsai H.-L. (2002). Antioxidant and pro-oxidant properties of ascorbic acid and gallic acid. Food. Chem..

[50] Bai J., Zhang Y., Tang C., Hou Y., Ai X., Chen X., Zhang Y., Wang X., Meng X. (2021). Gallic acid: Pharmacological activities and molecular mechanisms involved in inflammation-related diseases. Biomed. Pharmacother..

[51] Zhang L., Yue H., Zhang H., Xu L., Wu S., Yan H., Gong Y., Qi G. (2009). Transport stress in broilers: I. Blood metabolism, glycolytic potential, and meat quality. Poult. Sci..

[52] Pette D., Staron R.S. (1997). Mammalian skeletal muscle fiber type transitions. Int. Rev. Cytol..

[53] Wang T., Xu Y.Q., Yuan Y.X., Xu P.W., Zhang C., Li F., Wang L.N., Yin C., Zhang L., Cai X.C. (2019). Succinate induces skeletal muscle fiber remodeling via SUCNR1 signaling. EMBO. Rep..

[54] Li R., Li B., Jiang A., Cao Y., Hou L., Zhang Z., Zhang X., Liu H., Kim K.-H., Wu W. (2020). Exploring the lncRNAs related to skeletal muscle fiber types and meat quality traits in pigs. Genes.

[55] Zhang C., Luo J., Yu B., Zheng P., Huang Z., Mao X., He J., Yu J., Chen J., Chen D. (2015). Dietary resveratrol supplementation improves meat quality of finishing pigs through changing muscle fiber characteristics and antioxidative status. Meat. Sci..

